# Modulating platelet-activating factor by rupatadine attenuates gentamicin-induced nephrotoxicity in rats via NF-κB/caspase-3 and Nrf2/HO-1 signaling cascades

**DOI:** 10.1007/s00210-025-04327-0

**Published:** 2025-06-04

**Authors:** Reham H. Mohyeldin, Mahmoud Abdelnaser, Ehab E. Sharata, Al Shaimaa Mahmoud Kotb, Fatma F. Ali, Mina Ezzat Attya, Heba M. Tawfik, Mahmoud A. Elrehany, Remon Roshdy Rofaeil

**Affiliations:** 1 Department of Pharmacology & Toxicology, Faculty of Pharmacy, Deraya University, Minia, 61111 Egypt; 2 Deraya Center for Scientific Research, Deraya University, Minia, 61111 Egypt; 3 Department of Biochemistry, Faculty of Pharmacy, Deraya University, Minia, 61111 Egypt; 4 Medical Physiology Department, Faculty of Medicine, Minia University, Minia, 61519 Egypt; 5 Biochemistry, Molecular Biology, and Physiology Department, Faculty of Medicine, Mutah University, Al-Karak, Jordan; 6 Department of Pathology, Faculty of Medicine, Minia University, Minia, 61519 Egypt; 7 Department of Medical Pharmacology, Faculty of Medicine, Minia University, Minia, 61519 Egypt

**Keywords:** Gentamicin, Nephrotoxicity, Rupatadine, Nrf-2, Caspase-3

## Abstract

Gentamicin (GEN) is a commonly prescribed antibiotic for Gram-negative bacterial infections. One of the most common adverse consequences of it is renal damage which is developed in 30% of individuals receiving GEN for over 7 days. For the first time, we attempted to examine the reno-protective activity of rupatadine (RUP) on GEN-induced renal injury in rats. Renal damage was established by GEN in male Wistar rats. Histopathological analysis and kidney function panel were assessed. Levels of MDA, catalase, and SOD were detected using the colorimetric method. ELISA was utilized to assess the renal levels of IL-1β and TNF-α. qRT-PCR assessed mRNA levels of *Bax* and *Bcl-2*. Protein expression of Nrf-2, NF-κB, and caspase 3 were evaluated using Western blotting. GEN resulted in renal malfunction, high serum levels of cystatin C and BUN, increased renal levels of MDA, TNF-α, and IL-1β, decreased SOD and catalase activities, stimulated renal activation of NF-κB, and caspase 3 as well as inhibited the Nrf-2 protein expression, and upregulated *Bax* gene expression while it suppressed *Bcl-2* gene expression. Conversely, RUP administration markedly attenuated the nephrotoxicity of GEN. RUP suppressed the levels of the proinflammatory mediators, inactivated the renal NF-κB and caspase 3 proteins, declined renal mRNA levels of *Bax* gene, and upregulated the renal mRNA level of the *Bcl-2* gene. In conclusion, RUP mitigated GEN-caused renal damage by suppressing proinflammatory markers, mitigating apoptosis via repressing the intracellular PAF/NF-κB/caspase-3 pathway and upregulating Nrf2/HO-1 signaling cascades.

**Supplementary Information:**

The online version contains supplementary material available at 10.1007/s00210-025-04327-0.

## Introduction

Nephrotoxicity is a frequent side effect of several medicinal and diagnostic agents (Kim and Moon 2012). Twenty percent or more of patients admitted to hospitals with acute renal damage had drug-induced nephrotoxicity, with antibiotic-induced nephrotoxicity potentially reaching an incidence of 36% (Nolin and Himmelfarb 2010).

Infections caused by potentially lethal Gram-negative bacteria are often treated with the aminoglycoside antibiotic gentamicin (GEN) (Ali et al. 2005). A significant restriction of GEN is its potential to induce ototoxicity and tubular damage. In certain circumstances, these adverse consequences are so pronounced that the medication administration must be terminated (Lee et al. 2012). It is predicted that up to 30% of individuals receiving GEN for over 7 days exhibit evidence of nephrotoxicity (Lee et al. 2012; Cuzzocrea et al. 2002). The buildup of GEN in renal proximal convoluted tubules is linked to the specificity of GEN-induced nephrotoxicity (Nagai and Takano 2004). Moreover, 90% of administered gentamicin is not metabolized by the liver and is primarily eliminated intact via proximal renal tubules (Hu et al. 2017).

The mechanisms behind GEN caused renal damage are incompletely understood. Among the postulated pathways, oxidative stress, inflammation, and apoptosis are identified as primary contributors to nephrotoxicity resulting from GEN therapy (Tavafi and Ahmadvand 2011). It has been established that GEN promotes renal damage by boosting the kidney’s production of reactive oxygen species (ROS), resulting in apoptosis (Li et al. 2009; Kalayarasan et al. 2009). Conversely, GEN diminishes the efficacy of antioxidant defenses such as superoxide dismutase (SOD) and catalase (CAT) (Balakumar et al. 2010).

Aminoglycoside creates a complex with iron, and then the complex interacts with free oxygen radicals produced during arachidonic acid metabolism. This interaction activated nuclear factor-kappa B (NF-κB), leading to programmed cellular death and inflammation (Karasawa and Steyger 2011). NF-κB, the principal inflammatory factor, modulates several inflammatory mediators, such as tumor necrosis factor-alpha (TNF-α) and interleukin-6 (Brasier 2006; Oguz et al. 2015). NF-κB and its downstream proinflammatory cytokines are involved in pathogenesis of renal damage caused by GEN (Sahu et al. 2014; Adil et al. 2016). The up-regulation of NF-kB stimulates the expression of the pro-apoptotic protein Bax and decreases the expression of the anti-apoptotic protein Bcl-2, thereby facilitating apoptosis (Mahmoud et al. 2023).

Platelet-activating factor (PAF) is a powerful lipid mediator involved in several inflammatory processes, generated by nearly all cell types (Khalaf et al. 2022a). Multiple prior investigations have shown that elevated production and release of PAF significantly mediates the glomerular toxic effects of gentamicin (Rodriguez-Barbero et al. 1997; Martínez-Salgado et al. 2007; Al-Majed et al. 2002). Moreover, increased PAF production leads to NF-κB stimulation and its subsequent inflammatory markers (Choi et al. 2000).

One transcription factor that protects against a variety of toxins is nuclear factor E2-related factor 2 (Nrf2). In the promoter region of several genes that code for antioxidant enzymes, Nrf2 binds to the antioxidant response element and plays a crucial function in preserving cellular redox balance (Nguyen et al. 2003). Numerous studies indicated that the upregulation of Nrf2 confers protection against GEN-induced nephrotoxicity (Kalayarasan et al. 2009; Mahmoud 2017; Subramanian et al. 2015).

Rupatadine (RUP), a second-generation antihistaminic, is licensed for the treatment of allergic disorders and chronic urticaria. It has a long-acting blocking effect on the PAF receptor as well as the histamine-1 (H1) receptor (Khalaf et al. 2022a). Numerous investigations have demonstrated that RUP inhibits pro-inflammatory cytokines generated by PAF, including TNF-α and IL-6 production, to provide notable anti-inflammatory and antioxidant effects (Malavige et al. 2018; Didamoony et al. 2023). RUP mitigated inflammation in L-arginine-induced pancreatitis by suppressing the PAF/NF-κB/caspase 3 signaling cascade (Mohamed et al. 2022). Additionally, RUP alleviated 5-fluorouracil-induced hepatotoxicity via the modulation of the PAF/HO-1 pathway (Khalaf et al. 2022a). RUP attenuated diabetic nephropathy in rats via downregulation of the PAF/TNF-α signaling pathway (Hafez et al. 2020).

With a focus on the new involvement of PAF and histamine as inflammatory mediators in the pathophysiology of renal damage, the current work sought to explore the potential role of RUP, which is both mediators’ antagonist to attenuate GEN-related nephrotoxicity.

## Materials and methods

### Drugs

Rupatadine was procured from Sigma-Aldrich Chemical Co. (Missouri, USA), whereas GEN was sourced from EIPICO Pharmaceutical Company, Egypt.

### Animals and experimental design

Adult male Wistar albino rats weighing between 180 and 210 g were obtained from the Animal House of Nahda University in Beni-Suef, Egypt. Rats were provided with unrestricted access to food pellets and water one week before the experiment to facilitate their acclimatization to the laboratory setting. The subjects were maintained in a regulated environment with a lighting schedule of 12 h on and 12 h off, a temperature of 25 ± 2 °C and a humidity level of 45 ± 5% (Rofaeil et al. 2024). Approval of our study was provided by the institutional Ethical Committee of Deraya Center for Scientific Research (Deraya University, Minia, Egypt) following NIH Guidelines (NIH Publications No. 8023, revised 1978) and complied with the ARRIVE guidelines (Approval number: DCSR-010–024-22).

The rats were divided into four groups at random, with ten rats in each:

- Control group: rats received distilled water for 14 days.
- RUP 6 group: rats received RUP dissolved in distilled water at a dose of (6 mg/kg/day, p.o.) for 14 days (Mohamed et al. 2022; Ibrahim et al. 2022).
- GEN group: rats received daily i.p. injections of GEN (80 mg/kg) for 8 consecutive days (Al-Majed et al. 2002; Kumar et al. 2000).
- GEN/RUP 6: rats received RUP at a dose of (6 mg/kg/day, p.o.) for 14 days. Whereas, they administered GEN (80 mg/kg) starting from day 6 for 8 days (Mohamed et al. 2022; Ibrahim et al. 2022).

The dose of RUP was selected based on recent studies that illustrated its antioxidant and anti-inflammatory properties in experimental models of diabetic nephropathy, ulcerative colitis, and acute pancreatitis (Mohamed et al. 2022; Ibrahim et al. 2022). Furthermore, several studies have documented that GEN at 80 mg/kg for 8 days induced acute nephrotoxicity (Al-Majed et al. 2002; Kumar et al. 2000).

### Tissue isolation

On day 15, rats administered urethane at a dosage of (1.6 g/kg) by intraperitoneal injection. Blood was extracted from the abdominal aortas of rats and subsequently centrifuged for 15 min at 4000 g for biochemical analysis (Salama et al. 2020). Rats were euthanized by cervical dislocation, and the renal tissues were excised. The renal tissues were segmented into four sections, with the first and second sections immediately preserved at − 80 °C until homogenization for further biochemical analysis of inflammatory markers. The third section was stored at − 80 °C for quantitative reverse transcription polymerase chain reaction (qRT-PCR). The fourth piece was preserved in 10% formalin for histopathological and immunohistochemical evaluation.

### Biochemical assessment

#### Estimation of kidney function parameters

Serum BUN was assessed using a BUN kit (1,001,331, SPINREACT, Spain), serum cystatin-c was detected by ELISA cystatin-c kit (E-EL-R0304, Elabscience, USA), and serum creatinine was assessed by a creatinine kit (11,734, Biosystems, Spain) as directed by the manufacturer.

#### Oxidative stress markers determination

Renal superoxide dismutase (SOD) and malondialdehyde (MDA) were assessed by kits obtained from Biodiagnositic, Egypt (SD 25 21, MD 25 29, respectively). Renal catalase (E-BC-K031-S) was assessed by a kit obtained from Elabscience, USA as directed by the manufacturer.

#### ELISA assay of renal inflammatory markers, renal PAF, and renal HO-1

Renal contents of IL-1β (MBS265868) and TNF-α (MBS2507393) were assessed using ELISA kits (MyBioSource, USA) as directed by the manufacturer. Renal PAF was determined using an ELISA kit (ER1226, Fine test Co., Wuhan, China) as directed by the manufacturer. Renal HO-1 (E0676Ra) was determined using an ELISA kit (BT LAB, Zhejiang, China) as directed by the manufacturer.

### Quantitative real-time polymerase chain reaction

The Branson Digital Sonifer ultrasonic cell homogenizer (SFX 550, USA) was used to ultrasonically homogenize 100 mg of renal samples with 1 mL of TRIzol reagent. The total RNA quantity was determined, and the purity was measured using the A260/A280 ratio. qRT-PCR was carried out on RNA samples with purity scores of 1.7 or above. Using the Revert Aid First Strand cDNA Synthesis Kit (K1622, Thermofisher Scientific, USA), total RNA (in equal amounts) was converted into cDNA. Using single-stranded cDNA, real-time PCR was performed. Table 1 demonstrates the primer sequences. Using Thermo Scientific Maxima SYBR Green qPCR Master Mix (2X) (K0251, Thermofisher Scientific, USA) and Step One real-time PCR Detection System (4,369,074, Applied Biosystems, Singapore) were used to carry out the PCR process. We normalized the expression levels of each target gene in accordance with the expression levels of GAPDH mRNA (Rofaeil et al. 2024; Abdelnaser et al. 2023).

**Table 1:** Primer sequences

Primer	Sequences	
*Bax*	F	5′-CACGTCTGCGGGGAGTC-3′
	R	5′-TGTTGTCCAGTTCATCGCCA-3′
*Bcl-2*	F	5′-GGGCTACGAGTGGGATACTG-3′
	R	5′-GACCCCACCGAACTCAAAGA-3′
*GAPDH*	F	5′-CTCTCTGCTCCTCCCTGTTC-3′
	R	5′-CGACATACTCAGCACCAGCA-3′

### Western blotting

RIPA lysis buffer was used to homogenize renal tissue samples. After 5 min of treatment with loading buffer containing 2-mercaptoethanol, homogenates (30 µg of total proteins) were subjected to 12% sodium dodecyl sulfate–polyacrylamide gel electrophoresis (SDS-PAGE) and operating for 2 h at 100 V. After electrophoresis, proteins blotted onto PVDF membranes were blocked for 1 h in a tris-buffered saline (TBS-T) blocking solution containing 0.05% Tween-20 and 5% (w/v) non-fat milk. Using a Bio-Rad Trans-Blot SD Cell equipment (Bio-Rad, Hercules, CA, USA) (Mohyeldin et al. 2025), electrophoresis and electroblotting were performed. Incubation with primary antibodies including rabbit anti NF-κB p65 Rabbit (8242, cell signaling, USA), rabbit anti-Phospho-NF-κB p65 (3033, cell signaling, USA), rabbit anti-Nrf2 (20,733, cell signaling, USA) rabbit anti cleaved caspase-3 (9661, cell signaling, USA), and β-actin (sc-47778, Santa Cruz Biotechnology, CA, USA) were allowed overnight at 4 °C. In a blocking buffer solution, a secondary antibody, horseradish peroxidase-conjugated polyclonal immunoglobulin (1:5000) (#7074, Cell Signaling Technology Inc., MA, USA), was used. Immunoreactive proteins were detected using a luminous image analyzer (LAS-4000, Fujifilm Co., Tokyo, Japan) and the Chemiluminescence kit (GE Healthcare, Little Chalfont, UK) as directed by the manufacturer. The Image Processing and Analysis Java (ImageJ, 1.8.0_172) application was then used to accomplish densitometric analysis. Following normalization to the relevant β-actin levels, data was obtained in relation to a control group (Rofaeil et al. 2024).

### Histopathology

All the animal’s kidneys were taken out, promptly fixed in 10% formalin, processed, and stained with hematoxylin and eosin (standard procedure) (Bancroft and Layton 2013). The present study used a high-resolution digital camera and an Olympus (U.TV0.5XC-3) light microscope. Blind observation and evaluation were performed in a random order. Additionally, a semi-quantitative analysis was carried out using the Paller scoring method (Abdelnaser et al. 2024). Each kidney tissue segment slide’s parameters were scored, and the findings were presented as a five-point total score, with one point assigned for obvious tubular dilatation, two for degenerated renal tubules, and two for tubular necrosis in the renal tubule lumen (Li and Zhao 2024).

### Statistical analysis

The mean ± standard deviation (SD) was used to present all the data. The Shapiro–Wilk test was performed to test the normality of data, and the data were normally distributed because the p-value of all parameters was more than 0.05. As the data were normally distributed, a one-way analysis of variance (ANOVA) was used to analyze the results, followed by the Tukey–Kramer test to compare the significance difference between groups. GraphPad Prism® was utilized to perform statistical analysis. Significance levels were at *p* < 0.05.

## Results

All Comparisons among groups were conducted against the negative control group, as the data obtained from the RUP group did not exhibit any significant differences from that of the negative control group.

### RUP impact on kidney functions parameters

**Fig. 1:**
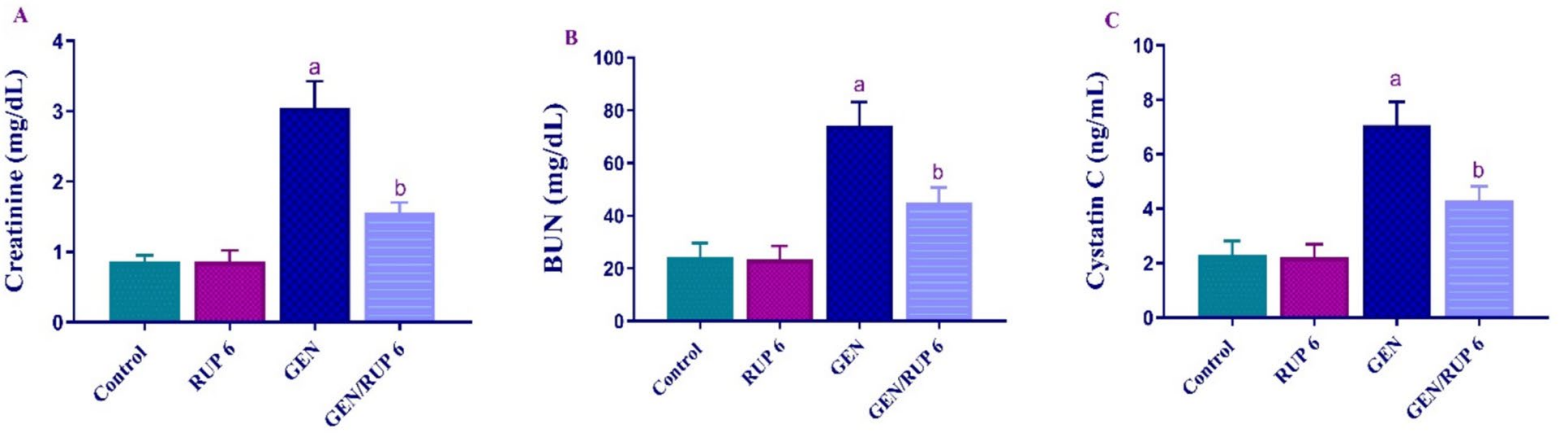
HYPERLINK"sps:id::fig1||locator::gr1||mediaobject::0"RUP effect on renal function panel in renal damage caused by GEN: creatinine levels (**A**), BUN levels (**B**), cystatin C (**C**). Each value (*n* = 6) is denoted by the mean ± S.D. Significance level: a; *p* < 0.05 vs control group, b; *p* < 0.05 vs GEN group. BUN: blood urea nitrogen, GEN: gentamicin, RUP: rupatadine

To determine the impact of RUP on kidney function, serum levels of creatinine, BUN, and cystatin C were evaluated. As shown in Fig. 1A–C, GEN markedly upsurged creatinine, BUN, and cystatin C serum levels to 3.55, 3.06, and 3.02 folds, respectively, in contrast to control group. However, GEN/RUP 6 group showed substantial diminished serum levels of creatinine, BUN, and cystatin C to 51.15%, 60.83%, and 59.86%, respectively, as compared to GEN group.

### RUP impact on renal oxidative stress markers

**Fig. 2:**
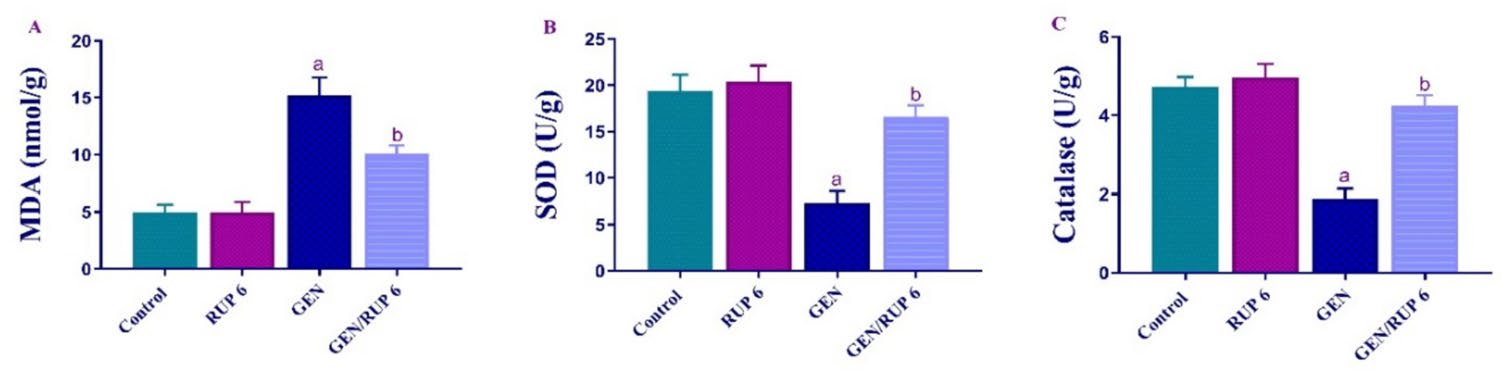
RUP effect on renal oxidative stress parameters in renal damage caused by GEN: MDA (**A**), SOD activity (**B**), catalase activity (**C**). Each value (*n* = 6) is denoted by the mean ± S.D. Significance level: a; *p* < 0.05 vs control group, b; *p* < 0.05 vs GEN group. GEN: gentamicin, MDA: malondialdehyde, RUP: rupatadine, SOD: superoxide dismutase

To evaluate the renal antioxidant impact of RUP, renal levels of MDA, SOD, and catalase were assessed. GEN group revealed a considerable elevation in the renal levels of MDA to 3.06-fold compared to control group. Conversely, GEN/RUP 6 group showed a considerable suppression of its renal levels to 66.21% in contrast to GEN group as depicted in Fig. 2A. Regarding SOD and catalase activity in the renal tissues, GEN markedly downregulated the activity of them to 37.78% and 39.92%, respectively compared to control group. Contrariwise, GEN/RUP 6 group demonstrated a significant increase in the activity of SOD and catalase to 2.27 and 2.24 folds, respectively in the renal tissues as demonstrated in Fig. 2B, C.

### RUP impact on renal PAF

**Fig. 3:**
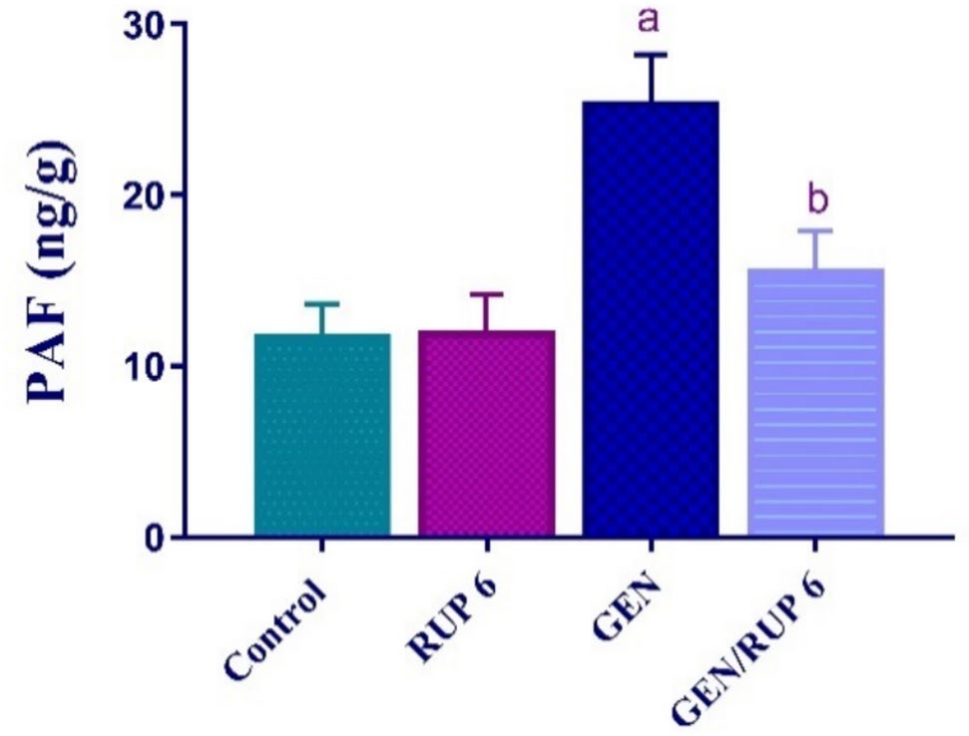
RUP effect on renal PAF in renal damage caused by GEN. Each value (*n* = 6) is denoted by the mean ± S.D. Significance level: a; *p* < 0.05 vs control group, b; *p* < 0.05 vs GEN group. GEN: gentamicin, PAF: platelet activating factor, RUP: rupatadine

To assess the renal RUP anti-inflammatory impact, the renal level of PAF was evaluated. As illustrated in Fig. 3, GEN dramatically inclined PAF renal levels to 2.15-fold relative to control group. In contrast, GEN/RUP 6 group showed a dramatic decrease in its renal level to 61.63% compared to GEN group.

### RUP impact on renal inflammatory markers

**Fig. 4:**
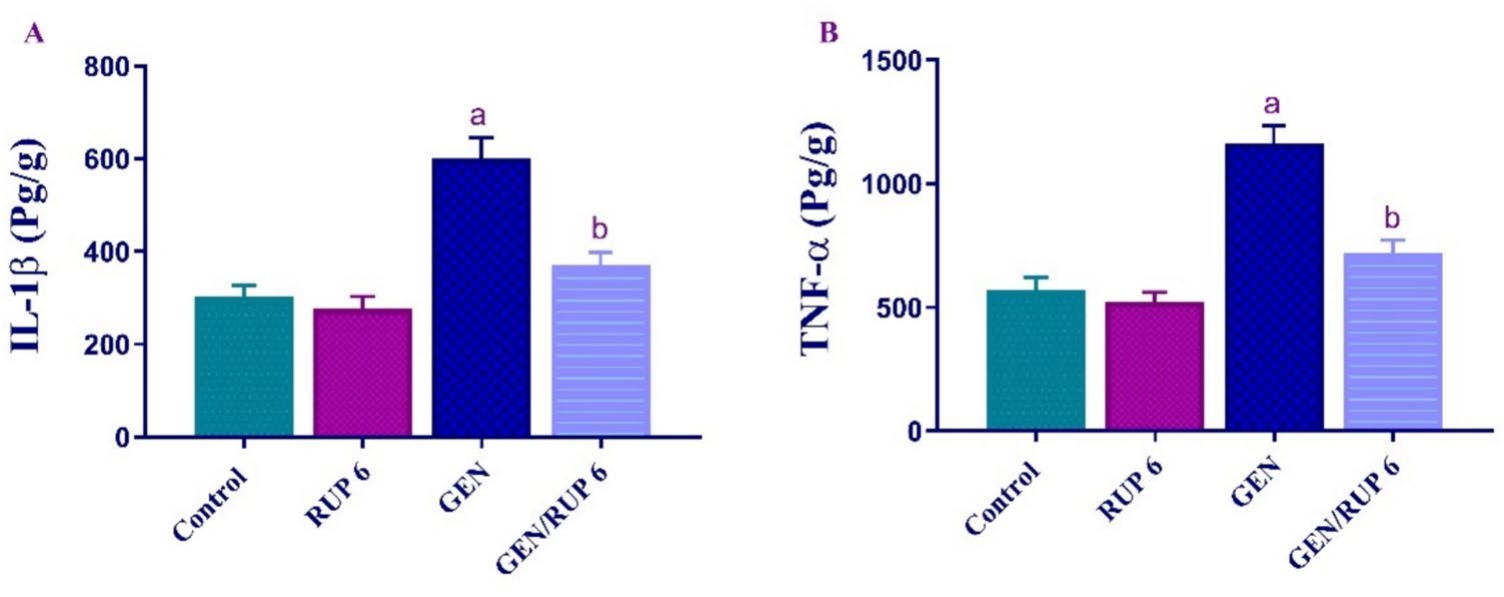
RUP effect on renal proinflammatory cytokines in in renal damage caused by GEN: IL-1β (**A**) and TNF-α (**B**) levels. Each value (*n* = 6) is denoted by the mean ± S.D. Significance level: a; *p* < 0.05 vs control group, b; *p* < 0.05 vs GEN group. GEN: gentamicin, IL-1β: interleukin 1 beta, RUP: rupatadine, TNF-α: tumor necrosis factor-alpha

To assess the renal anti-inflammatory impact of RUP, the renal level of IL-1β and TNF-α were evaluated. Figure 4A–B showed that GEN considerably increased the renal levels of IL-1β and TNF-α to 1.98 and 2.04 folds, respectively compared to the control group. Conversely, GEN/RUP 6 group illustrated a significant downregulation of their renal levels to 61.72% and 62.01%, respectively compared to GEN group.

### RUP impact on renal HO-1

**Fig. 5:**
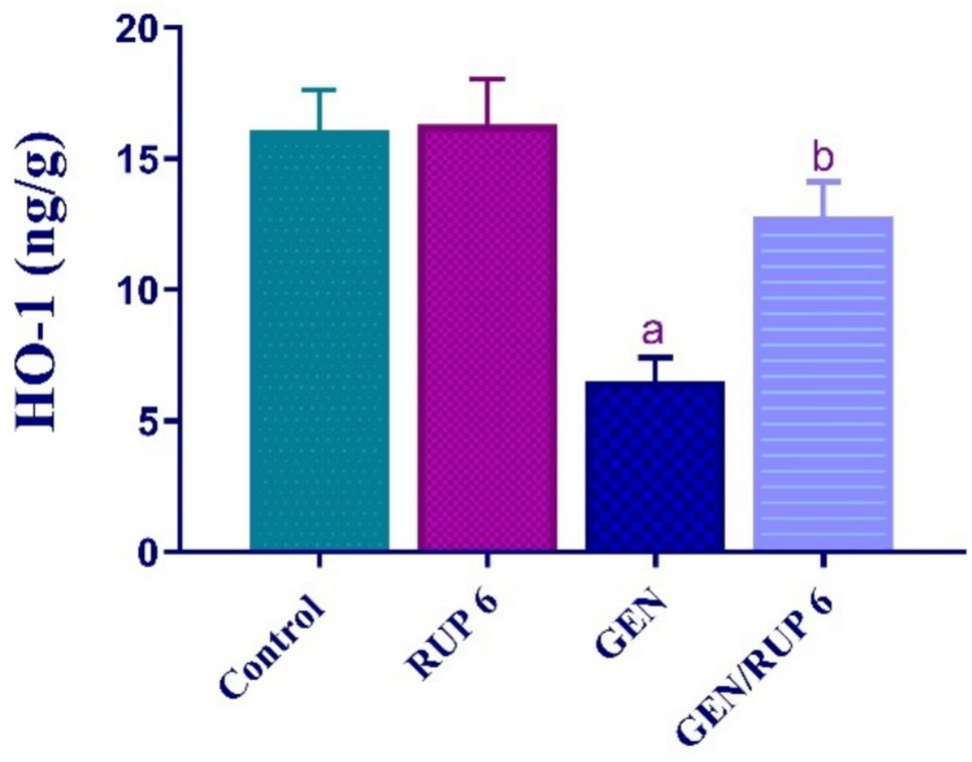
RUP effect on renal HO-1 in renal damage caused by GEN. Each value (*n* = 6) is denoted by the mean ± S.D. Significance level: a; *p* < 0.05 vs control group, b; *p* < 0.05 vs GEN group. GEN: gentamicin, HO-1: heme-oxygenase-1, RUP: rupatadine

To assess the ability of RUP to stimulate the antioxidant response elements the renal levels of HO-1 were determined. As depicted in Fig. 5, GEN markedly decreased the renal levels of HO-1 to 40.50% compared to control group. In contrast, renal HO-1 level was dramatically upsurged in GEN/RUP 6 group to 1.96-fold in comparison to untreated rats.

### RUP impact on renal Bax and Bcl-2 gene expression

**Fig. 6:**
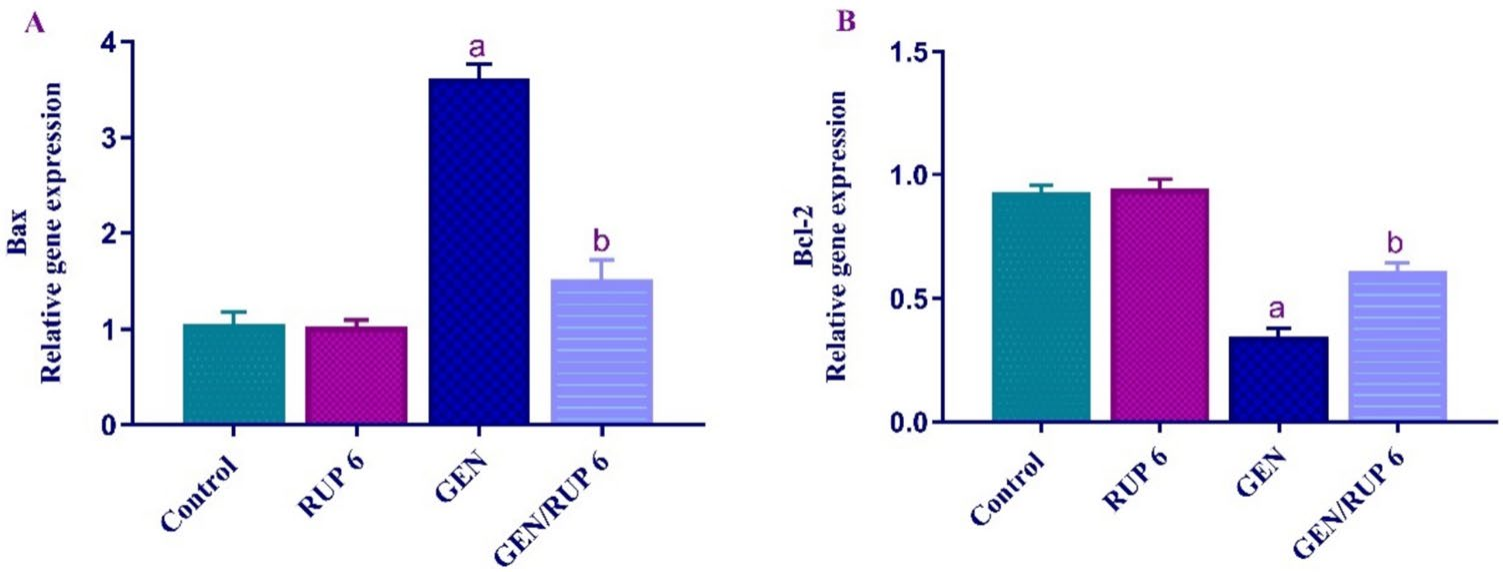
RUP effect on the renal expression of *Bax* (**A**) and *Bcl-2* (**B**) genes in renal damage caused by GEN. Expression was adjusted to GAPDH gene expression and expressed relative to the control group. Each value (*n* = 6) is denoted by the mean ± S.D. Significance level: a; *p* < 0.05 vs control group, b; *p* < 0.05 vs GEN group. Bax: (Bcl-2)-associated X, Bcl-2: B-cell lymphoma-2, GEN: gentamicin, RUP: rupatadine

To evaluate the RUP impact on the imbalance in the pro-apoptotic and anti-apoptotic parameters, the renal mRNA levels of *Bax* and *Bcl-2* were determined. Figure 6A, B depicted that GEN dramatically upregulated the renal gene expression of *Bax* to 3.44-fold*,* whereas considerably downregulated the renal gene expression of *Bcl-2* to 36.93% compared to control group. Conversely, GEN/RUP 6 group exhibited a significant suppression in the renal *Bax* gene expression to 41.96% and a marked stimulation of renal *Bcl-2* gene expression to 1.78-fold compared to GEN group.

### RUP impact on renal Nf-κB p-P65, cleaved caspase-3 and Nrf-2 protein expression

**Fig. 7:**
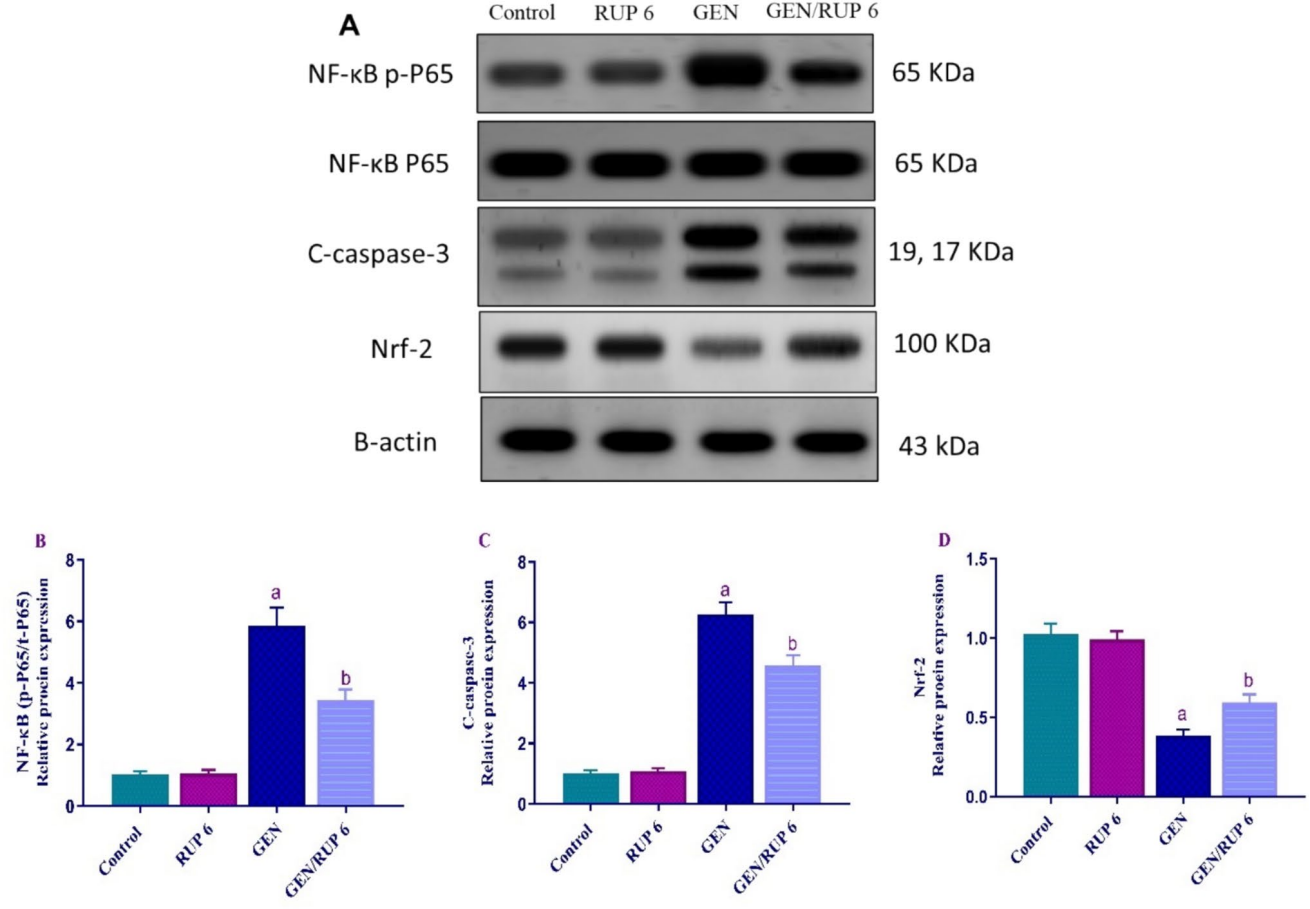
RUP effect on the renal expression of Nf-κB p-P65, cleaved caspase-3, and Nrf-2 proteins. **A** Representative western blots of Nf-κB p-P65, cleaved caspase-3, Nrf-2, and β-actin proteins in different groups. **B**–**D** After normalizing the bands in (**A**) to the appropriate internal control β-actin, densitometric analysis was utilized to determine protein expressions as fold change compared to control rats. Each value is denoted by the mean ± S.D*.* Significance level: a; *p* < 0.05 vs control group, b; *p* < 0.05 vs GEN group. GEN: gentamicin NF-κB: nuclear factor kappa B, Nrf-2: nuclear factor E2-related factor 2, RUP: rupatadine

To evaluate the anti-apoptotic impact of RUP in the renal tissues, the renal protein expression of Nf-κB p-P65, cleaved caspase-3 and Nrf-2 was detected using western blotting. As depicted in Fig. 7A–C, GEN significantly stimulated the activation of renal Nf-κB p-P65 protein to 5.79-fold and markedly increased the renal c-caspase 3 protein expression to 6.20-fold compared to control group. Conversely, GEN/RUP 6 group showed dramatic attenuation in their levels to 58.75% and 73.01%, respectively compared to the GEN group. Moreover, the renal protein levels of Nrf-2 were dramatically suppressed to 37.47% after GEN administration. However, GEN/RUP 6 group displayed a marked upregulation of its renal levels to 1.55-fold compared to GEN group as shown in Fig. 7D.

### RUP impact on renal histopathology

**Fig. 8:**
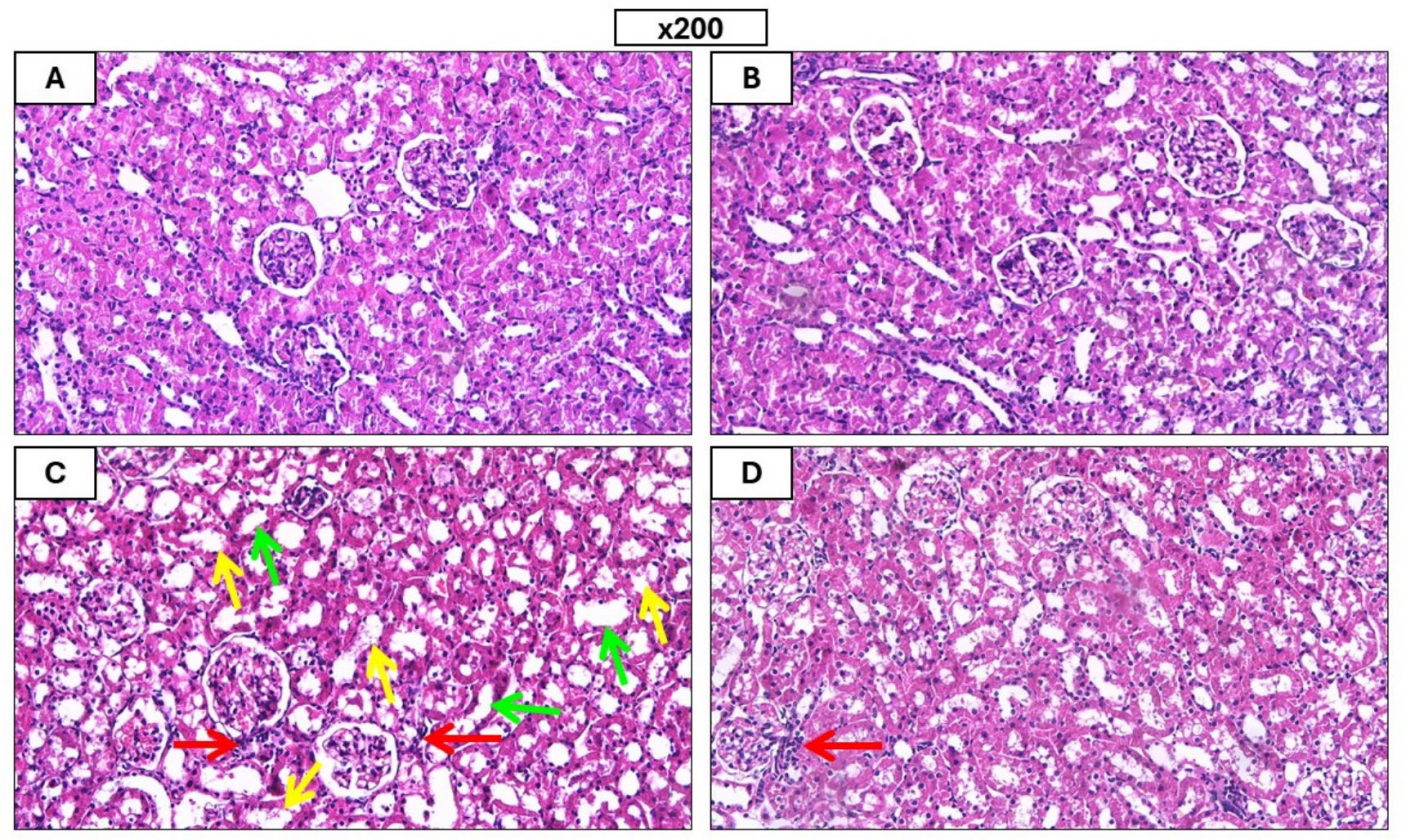
Histopathological alterations in kidney tissues. Images of the kidneys from several groups of rats (stained with H&E at 200x): control group (**A**), RUP 6 group (**B**), GEN group (**C**), GEN/RUP 6 group (**D**). Red arrow: inflammatory cells, green arrow: degenerated renal tubules, yellow arrow: vacuolar degeneration and necrotic tubular epithelial cells

**Fig. 9:**
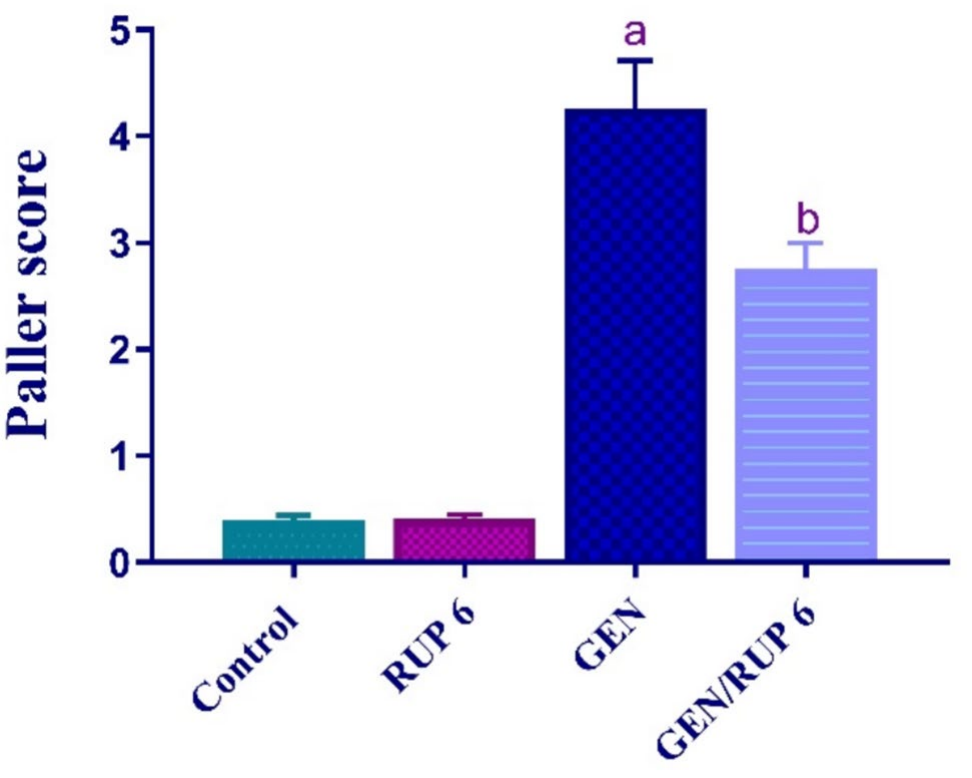
Paller score of renal damage caused by GEN. Each value (*n* = 6) is denoted by the mean ± S.D*.*Significance level: a; *p* < 0.05 vs control group, b; *p* < 0.05 vs GEN group. GEN: gentamicin, RUP: rupatadine

In contrast to control rats, which displayed normal renal tissues, the renal tissues of GEN rats displayed a dramatic elevation in inflammation, shedding of the brush edges of renal tubules, vacuolar degeneration, necrotic tubular epithelial cells, and evident tubular dilatation as shown in Fig. 8A–D. In contrast, Fig. 9 shows that GEN/RUP 6 group demonstrated a significant improvement in these changes.

## Discussion

GEN, an aminoglycoside antibiotic, has long been recognized for its efficacy against a wide range of infections primarily caused by Gram-negative pathogens; however, its nephrotoxic potential poses significant clinical challenges (Germovsek et al. 2017). Previous studies have thoroughly documented that renal impairment associated with gentamicin typically develops after a treatment period of 5 to 8 days, especially at doses ranging from 80 to 150 mg/kg; this presents significant limitations on its clinical use (Al-Majed et al. 2002; Kumar et al. 2000; Karahan et al. 2005). Consequently, it is imperative to safeguard patients from the detrimental impact of gentamicin on kidney cells and ensure their safety throughout the treatment protocol.

To the best of the authors’ knowledge, this study presents innovative findings that underscore the potential molecular mechanisms through which RUP alleviates gentamicin-induced nephrotoxicity targeting PAF/NF-κB/caspase-3 concomitant with modulation of Nrf2/HO-1 signaling pathway, which resulted in inhibition of oxidative, inflammatory and apoptotic signaling mechanism.

Several studies show that the administration of GEN is associated with glomerular function impairment, which is demonstrated by a decrease in renal functions, which is evidenced by a rise in serum urea and creatinine levels (Abdelnaser et al. 2024; Li and Zhao 2024).

Following GEN, all rats exhibited a reduction in glomerular filtration, as seen by increased blood levels of cystatin-C, BUN, and creatinine, which are considered the most sensitive indicators for detecting kidney damage in the experimental trials. Previous investigations have shown that oxygen-free radicals are significant mediators of GEN-induced nephrotoxicity (Randjelovic et al. 2012). Consequently, a primary strategy used to mitigate GEN-induced nephrotoxicity is the utilization of medications with strong antioxidant capabilities. The disturbed redox system in renal tissue was assessed by a significant rise in lipid peroxidation end product MDA accompanied with a reduction in CAT and SOD enzymatic activity relative to the control group of rats. Fortunately, the administration of RUP exhibits a significant diminution in serum cystatin-c, BUN, and creatinine serum concentration which reflects undeniable enhancement in renal function when compared to the model group of rats. Alongside improvement in kidney function, RUP completely reversed the highest level of MDA shown in the GEN-medicated group of rats paralleled by the marked increase in SOD and CAT enzymatic activity. RUP antioxidant impact has been proved previously in many experimental models for instance in testicular ischemia/reperfusion injury, diabetic nephropathy rats’ model, and as well as allergic rhinitis patients (Hafez et al. 2020; Abdel-Aziz et al. 2024; Kahveci et al. 2020).

The molecular mechanism underlying gentamicin-induced renal toxicity is complex and multifactorial, involving various pathways that contribute to renal tissue toxicity (Mingeot-Leclercq and Tulkens 1999). However, the pathophysiology of GEN-induced nephrotoxicity involves an imbalance between antioxidants and oxidants. Previous research demonstrates a close correlation between the pathophysiology of GEN-inducing nephrotoxicity and an increase in PAF production in glomeruli (Harisa 2012). We hypothesized that regulating the PAF/NF-κB/caspase-3 pathway and its crosslinking with the Nrf-2/HO-1 pathway could indeed offer significant protective effects against GEN-induced nephrotoxicity.

In order to elucidate the hypothesized new mechanistic insights into RUP reno protective role against GEN induced acute renal injury PAF, NF-KB, Nrf-2, and HO protein levels were measured to discover its impact on mitigating inflammatory cytokines and apoptotic processes.

PAF is a lipid mediator that is implicated in a variety of renal disorders, affecting the kidney through immune, inflammatory, and vasoactive mechanisms (Bertani et al. 1987). PAF results in a decline in glomerular filtration rate (GFR) due to arteriolar vasoconstriction and/or an effect on mesangial cell contraction. Thus, PAF appears to play a role in certain types of acute renal failure. The production of PAF is influenced by oxidative stress and ROS (Yamakawa et al. 2000). Furthermore, The involvement of PAF in the development of ARF is additionally corroborated by the impact of the specific antagonist of PAF “BN-52021.” Intriguingly, the study reported that PAF blocker markedly blunt the fall in GFR confirmed by attenuation of the tissue necrosis induced by GEN (López-Novoa 1999).

In alignment with the abovementioned findings our results showed that GEN-treated group rats exhibit the highest level of PAF protein level with concomitant increase in cellular inflammatory cytokines expression TNF-α, IL-1β, and IL-6, in addition to an increase in lipid peroxidation level as mentioned above which confirm the buildup of cellular ROS. Fortuitously, RUP medicated group of rats exhibited an undeniable decline in the level of PAF protein expression accompanied by significant dampening in proinflammatory cytokines which can support the putative amendatory effect of RUP against GEN-induced ARI. As per Mohamed et al. (2022) results, RUP confers intestinal mucosal protection against 5-FU-induced intestinal mucositis concurrently with an indisputable decrease in PAF, MDA, TNF-α, IL-1β, and IL-6 with respect to intoxicated animals (Mohamed and Mohammed 2022).

Moreover, A prior study confirmed that there is an increase in the transcription of proinflammatory cytokines, particularly. TNF-α and IL1β cause further activation of PAF production and secretion by mesangial cells which exacerbate the injury of the renal tubules (Reznichenko and Korstanje 2015).

In order to clarify the molecular mechanism involved in the protective effect of RUP against GEN induced ARI so the cross talk between PAF, NFK-B, and Nrf-2 was chased in the current study. It was evident that once activation of the PAF-R downstream signaling cascade turned on and enhances cell apoptosis via activation of the NF-KB/Ca +/calcineurin signaling pathway which showing a loop of amplification of the inflammatory process through transcription of pro inflammatory cytokines such as TNF-, IL6, and IL-1B (Martinez-Salgado et al. 2007; Khallaf et al. 2023). Furthermore, the upregulation of NF-κB due to PAF signaling exacerbates tubular cell apoptosis through the enhancement of pro-apoptotic protein expression like Bax while decreasing anti-apoptotic factors such as Bcl-2 (Zonouz et al. 2024).

The present investigation revealed a considerable increase in the NF-κB protein, as well as a simultaneous rise in the levels of proinflammatory cytokines TNF-α, and IL-1β in the GEN-treated group. These findings corroborate prior research that establishes the interaction between PAF-R activation, NF-κB transcription factor, and the activation of the apoptotic cascade (Ozbek et al. 2009). Conversely, the administration of RUP significantly decreased the elevated level of NF-κB protein in addition to a significant decrease in the abovementioned proinflammatory cytokines production. This beneficial effect of RUP can be attributed to blocking PAF receptors and consequently IL-β/IL-6/IL-TNF-α axis.

The interaction between Nrf2 and NF-κB has emerged as a significant target in kidney disease, since its modulation may reduce the pro-inflammatory cascade and boost the antioxidant response (Jaiswal 2004). Numerous cytoprotective proteins and enzymes that aid in cellular defense against ROS and prevent NF-kB from moving to the nucleus are expressed when the Nrf2 pathway is activated. Additionally, Nrf2 overexpression improves antioxidant responses by increasing HO-1 expression, which raises Bcl2 levels and lowers Bax and caspase 3 to prevent ROS production and cellular death (Mundkar et al. 2022; Abdelnaser et al. 2025). Our study reported a massive decrease in NF-kB expression parallel with enhancement in Nrf2 and HO-1 in comparison to the intoxicated group of rats. These results explain the decrease in the level of BAX and caspase-3 protein expression aligns with the increase in the expression of Bcl-2 in RUP-treated group of rats. The results are consistent with previous studies which indicated that RUP has a perfect safety profile in different clinical studies (Khalaf et al. 2022b) and has demonstrated protection against pulmonary fibrosis by inhibiting the PAF-mediated pathway, which involves a reduction in the infiltration of inflammatory cells into the lung, resulting in a subsequent reduction in the release of inflammatory cytokines in the inflamed lung (Lv and Wang 2013).

## Conclusion

**Fig. 10:**
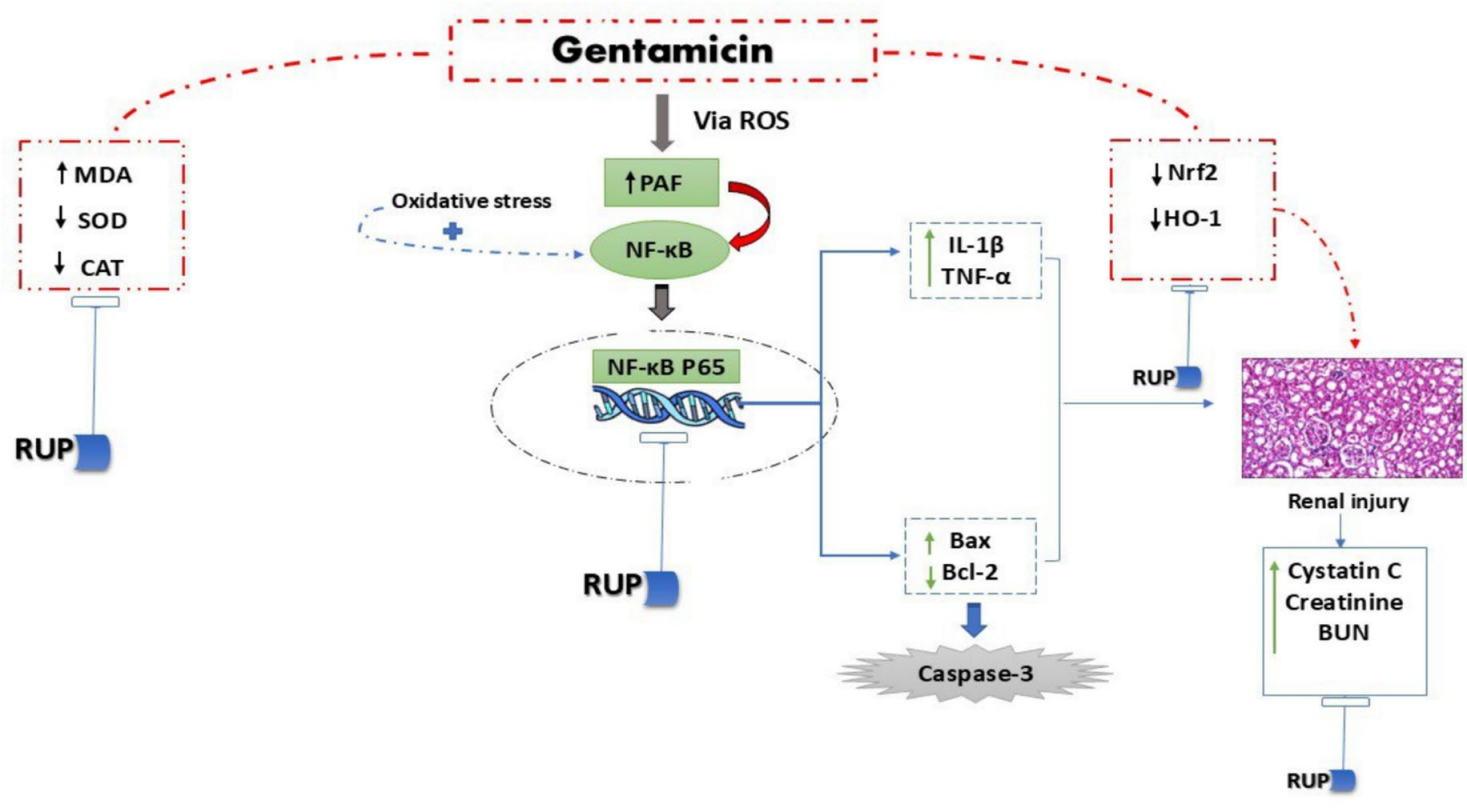
Figure illustrating the mechanisms of GEN induced renal injury and the protective impact of RUP

In conclusion, the present work offers new understanding of the molecular pathways through which RUP mitigates GEN-induced nephrotoxicity. By targeting the PAF/NF-κB/caspase-3 pathway and modulating the Nrf2/HO-1 signaling pathway, RUP effectively reduces oxidative stress, inflammation, and apoptosis in renal tissues as depicted in Fig. 10. This dual action underscores the potential of RUP as a therapeutic agent to protect against GEN-induced renal injury. In this study, we posit that combining GEN with RUP may provide a strategic advantage in managing infections, especially in patients at risk of nephrotoxicity. By leveraging rupatadine’s anti-inflammatory and nephroprotective properties, clinicians may enhance patient safety without compromising therapeutic efficacy, ultimately leading to better management of infections and preservation of renal function. Finally, further clinical studies are warranted to validate these benefits and optimize treatment protocols.

## Supplementary Information

Below is the link to the electronic supplementary material.

Supplementary file1 (PDF 234 KB)

## Data Availability

All data produced or examined during this investigation are incorporated in this published article.
