# Supplementary material for: Modulating platelet-activating factor by rupatadine attenuates gentamicin-induced nephrotoxicity in rats via NF-κB/caspase-3 and Nrf2/HO-1 signaling cascades
Source: Naunyn Schmiedebergs Arch Pharmacol. 2025 Jun 4;398(12):17135–46. doi: 10.1007/s00210-025-04327-0 (PMC12678517; doi:10.1007/s00210-025-04327-0)
Supplement: Supplementary file 1 — Supplementary file1 (PDF 234 KB) [file 210_2025_4327_MOESM1_ESM.pdf]

Cleaved caspase-3

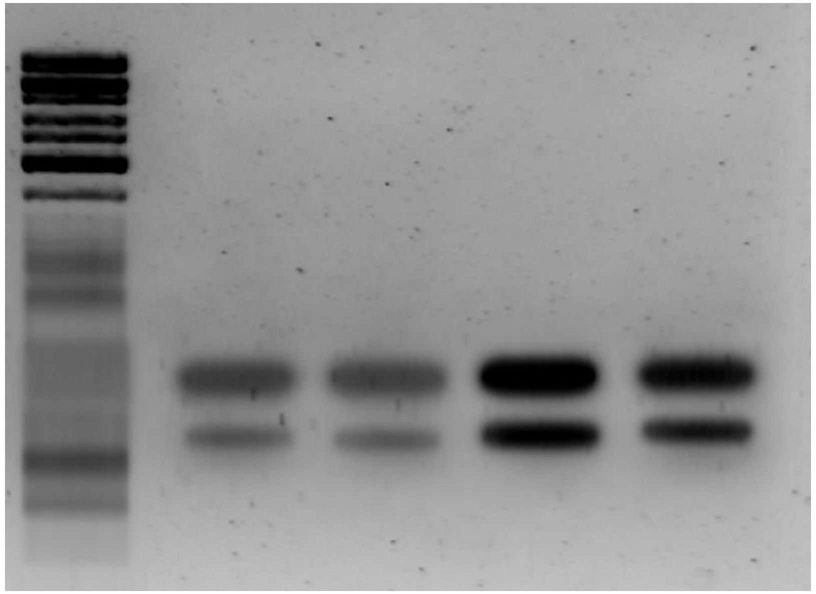

C-caspase-3

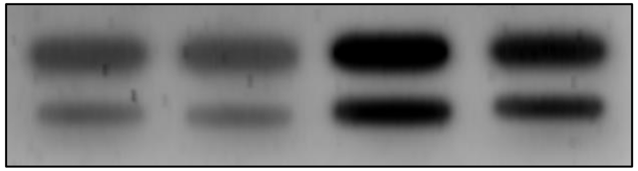

19, 17 KDa

NF-κB p-P65

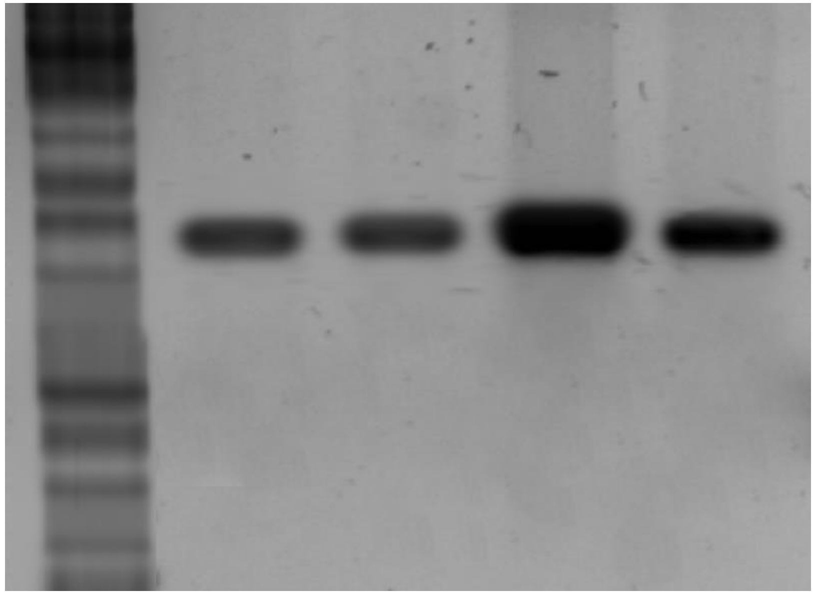

NF-κB p-P65

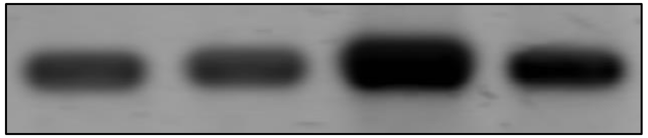

65 KDa

NF-κB P65

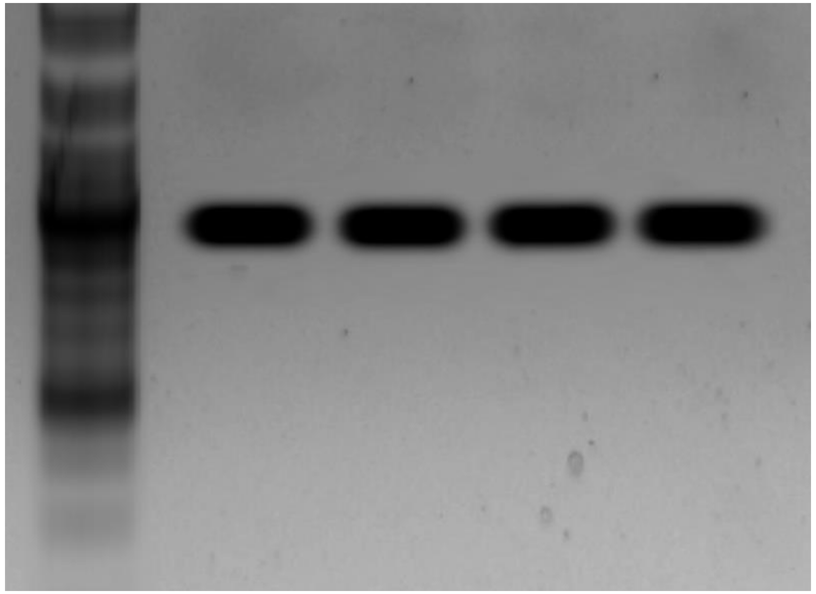

NF-κB P65

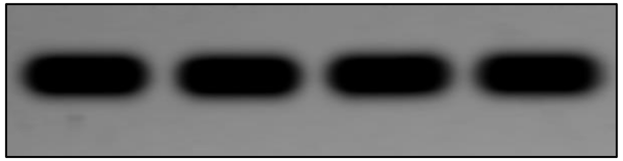

65 KDa

Nrf-2

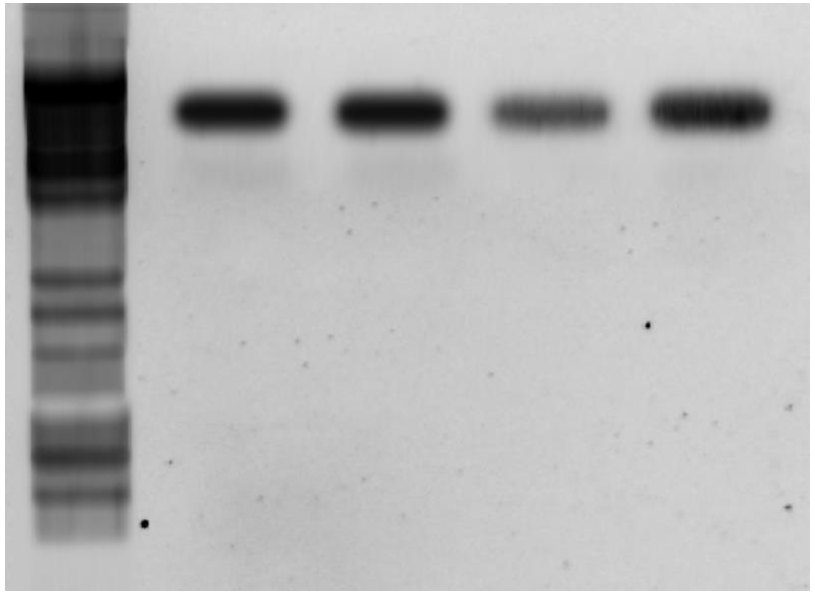

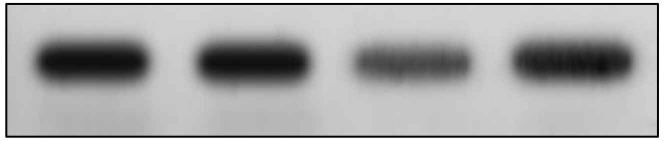

B-actin

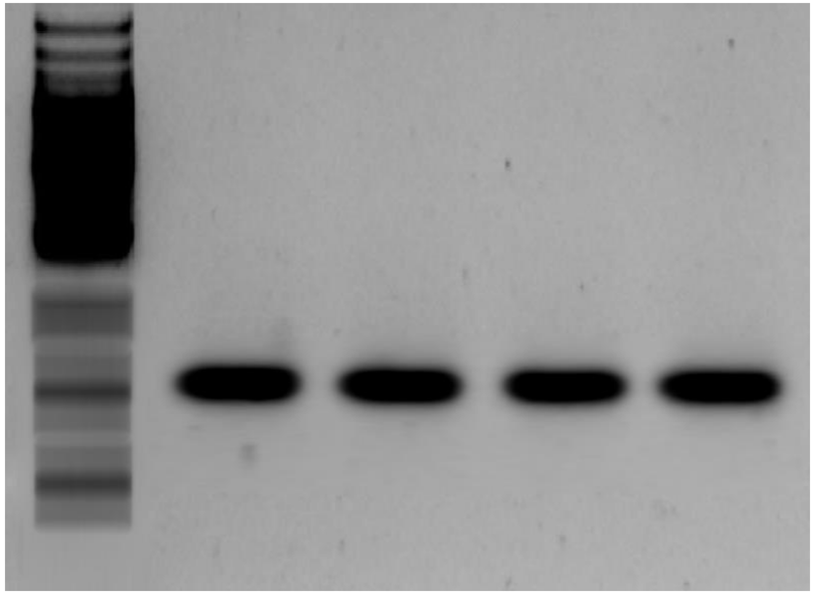

B-actin

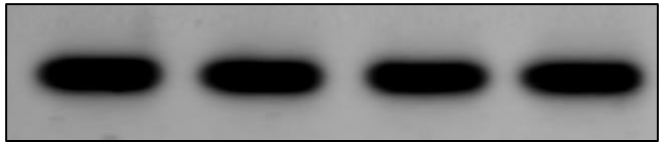

43 kDa
